# Policy brief Belgian EBCP mirror group ‘prevention’ and ‘early detection & screening’ in cancer

**DOI:** 10.1186/s13690-024-01368-4

**Published:** 2024-08-30

**Authors:** Cindy Simoens, Gabrielle Schittecatte, Belgian EBCP mirror group ‘Prevention and Early detection & Screening’

**Affiliations:** Sciensano, Cancer Centre, Brussels, Belgium

**Keywords:** Prevention, Early detection, Screening, Dara linkage, Emerging technologies, Policy Recommendations

## Abstract

The availability and linkage of comprehensive data on cancer mortality, incidence, screening and vaccination (HPV/HB) are essential for monitoring the process and impact of all screening and vaccination programmes, and to generate evidence to improve prevention policies. Linkage of data registries will allow better exploitation of real-world data. Simplification of data sharing for epidemiological research is of utmost importance. Human, as well as technical/technological and legal/regulatory barriers need to be tackled in Belgium to aid actors involved in screening, to facilitate organised linkage of data. Rapid advances in technologies for early detection and screening for a broad range of cancer types provide significant opportunities. Such tests are not yet ready for adoption in screening programmes. Emerging evidence should be monitored to ensure that promising innovations can transition to implementation studies in a timely manner. More and sustained research is needed to generate the necessary scientific relevance for new screening techniques in relevant cancer types.

**Supplementary Information:**

The online version contains supplementary material available at 10.1186/s13690-024-01368-4.

## Background

Two key pillars of the Europe’s Beating Cancer Plan (EBCP) are ‘Prevention’ and ‘Early detection’, with potential for actors to generate great added value. Prevention is more effective than any cure, both at the individual and the societal level. The European Code Against Cancer has proposed 12 ways to reduce cancer risk in Europe [1]. Moreover, there is the European commitment to respond to the WHO initiative to eliminate cervical cancer [2, 3].

Similarly, early detection offers one of the best chances of beating cancer and saving lives. Under the EBCP, early detection of cancer is promoted through improving access, overall quality and diagnostics, while supporting Member States (MS) to ensure that 90% of the EU population who qualify for breast, cervical and colorectal cancer (CRC) screenings are offered screening by 2025. To achieve this, a new EU-supported Cancer Screening Scheme will be proposed. Extending targeted cancer screening to include additional cancers, such as prostate (no preventable cause identified), lung and gastric cancer, will also be considered [4].

## Main text

### Issue Overview

#### Comprehensive registration & data linkage

Data linkage brings information from different sources together to create a new, richer dataset; providing chronological sequences of events leading up to, or subsequent to, cancer diagnosis. At a macro level, linkage can inform health policy stakeholders and orient research. Registries that can be linked in Belgium include:

- ➢ **The InterMutualistic Agency (IMA-AIM)** manages a health administrative database, providing data on reimbursed vaccination, screening indicators trend, cost burden of cancer, longitudinal health consumption, etc. It is also a repository of information needed for implementation of population-based screenings, such as eligible groups, follow-up, and failsafe.
- ➢ **The Belgian Cancer Registry (BCR)** monitors the burden of cancer and all (cyto-histo-pathological) samples relating to cancer types with organised screening programmes. BCR contributes to efficient organization and evaluation of screening programmes through established linkage with external data sources (e.g. population data including vital status, data from IMA-AIM on diagnostic procedures and treatment, cancer screening data).
- ➢ **Screening registries** allow the monitoring of screening performance indicators and define the list of eligible people needing invitations/reminders. Currently, 3 regionally organised screening centres exist: *Centrum voor Kankeropsporing (CvKO)* in Flanders, *Centre Communautaire de Référence pour le dépistage des cancers (CCR)* in Wallonia, and *BruPrev* in Brussels.
- ➢ **Vaccination registries** allow to monitor HPV/HB vaccine use and vaccination coverage if registration is exhaustive and vaccination records are unique. Through exchange between multiple registries, the functionality of each can be improved and a higher level of completeness can be reached. Both *Vaccinnet (Flanders) and the Platform e-vax (Fédération Wallonie-Bruxelles*) are ordering and distribution systems for vaccines that are made available by health authorities to health care workers in the context of its programmatic vaccination policy. These ordering systems are linked to a registration system for vaccinations. However, registration is not obligatory in *e-vax* and therefore not complete, creating large differences between both regional registries.

Comprehensiveness is a very important quality characteristic of the registries. Moreover, individual unique identifiers and citizen consent are indispensable for adding longitudinal follow-up and linkage with other registries. In order to evaluate the impact and safety of preventive interventions, linkage between exhaustive vaccination, screening and cancer registries are needed in Belgium. However, sustained data linkage is not yet in place, not all registries are comprehensive, and some might have overlapping data.

#### Emerging technologies

Liquid biopsy (screening using blood, urine or breath) is emerging as a minimally invasive, highly specific technology for multiple cancers. Promising technologies to address early disease biomarkers from liquid biopsies include measuring proteomics, metabolomics and exhaled volatile organic compounds (VOCs). While these tests may not yet be prepared for integration into national or regional screening programs, it is critical to monitor the evolving evidence-base. This vigilance ensures that promising innovations are promptly advanced into implementation studies, not only in Belgium, but across the EU. Artificial Intelligence (AI) algorithms can also help to streamline screening logistics and reduce pathology and radiology bottlenecks in the future [5].

Strategies based on risk stratification are developing rapidly. The identification of population subgroups with a higher risk of cancer, using polygenic risk scores (PRS), could lead to more effective stratified cancer screening programmes. The importance of emerging technologies for the benefit of cancer prevention/early detection is growing. Belgium should take advantage of these innovations as more scientific evidence is generated.

## Projects and Gaps

### Comprehensive registration & data linkage

#### Vaccination

Different HPV vaccination programs exist in the three regions. In Wallonia and Brussels girls and boys aged 13–14 years or attending the second year of secondary school are offered vaccination, free for all born in 2006 or later. In Flanders, the vaccine is free for girls and boys in the first year of secondary education and those of the same age in special education [6]. No catch-up vaccination is offered. Data show large disparities in coverage between the Flemish and the French Community (91% versus 36%, respectively) [7, 8]. The regional difference in coverage may result in one part of Belgium having more HPV related cancers than the other part [9].

The PERCH project (PartnERship to Contrast HPV) [10], supports MS efforts to extend the roll-out of routine HPV vaccination of girls and boys to eliminate cervical cancer and other cancers caused by HPV in the coming decade. This joint action will support the exchange of validated best practices between the MS to ensure a consistent and efficient roll-out of HPV vaccination.

This project will result in optimised organisation of vaccination, improved vaccine coverage for Brussels and Wallonia, and improved access for vulnerable populations. It will also support improved data collection and monitoring options regarding vaccination coverage, to be linked with screening, cancer and mortality data. Comprehensive vaccination registration is not available for Belgium and linkage with the other data is still not routinely done in Belgium. This is urgently needed to follow up on the vaccination coverage. Without this linkage step it remains a challenge to evaluate the impact and safety of HPV vaccination.

#### Screening

Cancer screening is organised at the regional level in Belgium [11]. These regional levels are supported by centres of expertise in the area of screening (see Issue Overview - Comprehensive registration & data linkage), which each have their own monitoring system and databases. There is some collaboration, centralization and harmonization through BCRs collaboration with the 3 centres.

CanScreen-ECIS [12] supports the integration of cancer screening data provided by the cancer screening programmes into ECIS (European Cancer Information System), to facilitate the permanent monitoring of the screening programmes, including the performance indicators.

It improves the collection of screening data according to EU formats, enabling uniform documentation and reporting of screening coverage, processes and outcomes by region, reflecting actual coverage realised by existing programmes. Establishing a comprehensive overview of screening coverage facilitates targeted actions to optimise programmes, including understanding where to extend current screenings. It will also enable Belgian actors to learn from the best practices in Europe. Current linkage of regional data and BCR data allows some calculation and reporting. However, a sustained linkage and extension to other available data registries is a necessity.

#### Emerging technologies

**Fig. 1:**
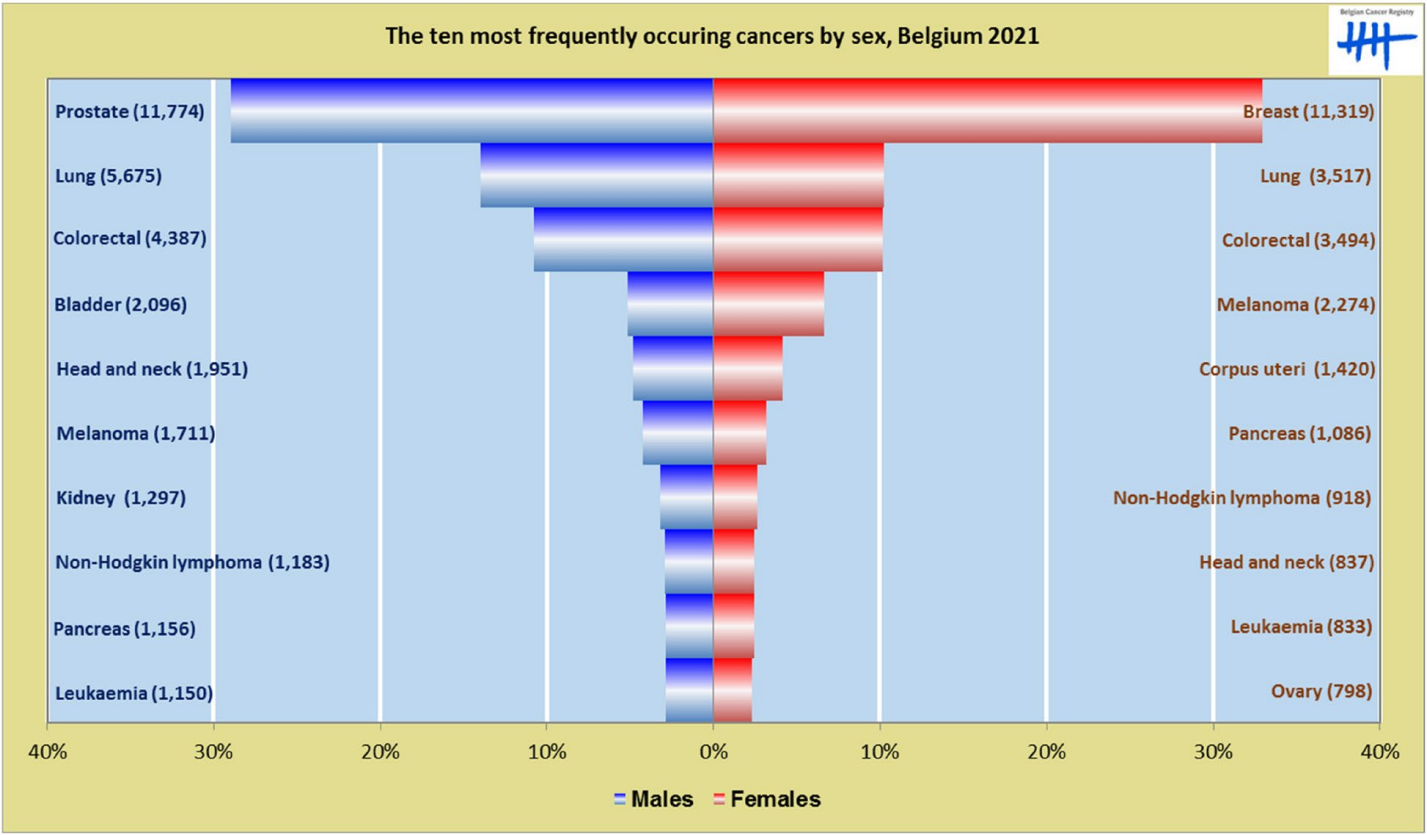
The ten most frequently occurring cancers by sex, Belgium 2021

Lung cancer is the most important cause of death by cancer in Belgium (*N* = 5.667; Crude mortality rate: 49.3/100.000 person years, in 2020 [13], and prostate cancer has the highest morbidity among Belgian men (Fig. 1) [14]. However, there are currently no organized screening programmes for prostate or lung cancer in Belgium.

SOLACE [15] and PRIASE-U [16] are two large scale European pilot studies for lung and prostate cancer screening, respectively. They will focus on risk stratification algorithms using Prostate-Specific Antigen testing and MRI imaging (prostate), low-dose CT imaging (lung), AI-based incidental findings, and automated workflows. Both pilots will develop concrete recommendations for MS on how to run effective lung/prostate cancer screening programmes.

The added value for Belgium can be found in the increased early stage findings with quick access to care pathways, improving staff experience and patient survival (or survivorship), and at a significant lower cost.

The ONCOSCREEN project [17] responds to the challenge of implementation of personalized CRC screening across the EU by developing a risk-based, population-level stratification methodology. It complements this methodology by ^a)^developing a set of novel, practical, and low-cost screening technologies with high sensitivity/specificity, ^b)^leveraging AI to improve existing methodologies for CRC screening, allowing for the early detection of polyps and provision of a personalized risk status stratification, and ^c)^providing a mobile app for self-monitoring and CRC awareness raising.

An Intelligent Analytics Dashboard will be developed for Belgian policy makers, facilitating effective policy making at regional and national levels by extending in-house (VITO) GIS technology with CRC screening and incidence data across EU.

The CAN.HEAL [18] consortium opens new perspectives to personalised risk assessment and targeted cancer prevention by developing tools and procedures to establish integration or alignment of population-based interventions based on genomics data obtained through massive parallel genome analysis of DNA/RNA. It will develop recommendations for a genomic risk assessment report based on PRS for physicians and genetic counsellors.

## Policy recommendations

### Comprehensive registration & data linkage

Availability and linkage of comprehensive data on vaccination, screening, cancer incidence and mortality are essential to monitor the processes and impact of both vaccination and screening programmes, and to generate evidence-base to optimise screening programmes. Linkage of registries is needed to allow better exploitation of real-world data.

The primary barriers to overcome, to support actors and facilitate linkage of data, are:

1. Human aspects (informed consent, governance, priorities)
2. Technical/technological aspects (interoperability, cyber-security, privacy)
3. Legal & regulatory aspects (GDPR compliance, ethical standards, national laws & regulations)

Of utmost importance is the simplification of procedures to share data for epidemiological research. The Belgian government should actively facilitate this, with clear and stable guidance, and minimal turnaround time for permissions; while respecting privacy regulations. Individual data linkage should be made a real possibility, not only a theoretical one.

## Emerging technologies for existing and new screening programmes

Ongoing EU projects focus on genetics, VOCs, metabolomics and AI for CRC screening, but further sustained research is needed to generate the necessary scientific evidence, including screening for other cancer types. For example, breast cancer, where genetic predisposition is of great importance [19], but also presymptomatic screening for high-risk genes to detect ovary, colon, uterus and prostate cancer for the broader population, not only high-risk individuals.

PRS represent a significant advance in breast cancer risk prediction, which is clinically useful when used in addition to existing risk models, that include clinical and lifestyle risk factors, and mammographic density as previously demonstrated. The best approach to implement PRS at a clinical setting on an individual level, is yet to be determined in the Belgian context. More research should focus on polygenic testing, not only to generate reliable scientific evidence, but also including how to communicate on the topic towards health professionals and patients, as well as how to tackle the ethical and data protection issues that arise with these type of tests.

In Belgium, a promising tool for early *Non-Invasive pre-symptomatic Cancer detection by liquid biopsy Testing* (NICT) based on AI of shallow whole genome sequencing cell-free DNA is under development. If confirmed, this innovation could provide a sensitive, cost-effective, minimally invasive method for non-invasive pan-cancer screening and early diagnosis. To do so, we call on government to support the following, in the clinical setting:

- ➢ standardising the detection process utilising a NICT platform
- ➢ pilot projects/RCT comparing NICT with established screening methods
- ➢ verifying the feasibility and cost-efficiency of NICT
- ➢ cost-effectiveness and cost-utility analyses evaluating the impact of a national pan-cancer screening program based on NICT.

AI and other new technologies should prove their clinical value before implementation in routine screening in CRC. This also holds true for establishing new screening strategies, such as skin cancer screening, to make it feasible for the general population. Further support for research is needed on these topics, in order to generate insights, particularly on how to feasibly reach and identify the target populations for new cancer type screenings at population level.

### Supplementary Information

Supplementary Material 1.

## Data Availability

Not applicable.

## Abbreviations

AI: Artificial Intelligence
BCR: Belgian Cancer Registry
CCR: Centre Communautaire de Référence pour le dépistage des cancers
CRC: Colorectal cancer
CvKO: Centrum voor Kankeropsporing
EBCP: Europe’s Beating Cancer Plan
IMA-AIM: Intermutualistic Agency
MS: Member States
NICT: Non-Invasive pre-symptomatic Cancer detection by liquid biopsy Testing
PRS: Polygenic risk scores
RCT: Randomised controlled trial
VITO: Vlaamse Instelling voor Technologisch Onderzoek (Flemish Institute for Technological Research)
VOCs: Volatile organic compounds
