# Supplementary material for: Policy brief Belgian EBCP mirror group ‘prevention’ and ‘early detection & screening’ in cancer
Source: Arch Public Health. 2024 Aug 30;82(Suppl 1):144. doi: 10.1186/s13690-024-01368-4 (PMC11363375; doi:10.1186/s13690-024-01368-4)
Supplement: Supplementary file 1 — Supplementary Material 1. [file 13690_2024_1368_MOESM1_ESM.docx]

## Members of the EBCP Mirror Group ‘Prevention & Early Detection’

| Last Name | First Name | Institution |
| --- | --- | --- |
| ALBARANI | Valentina | NCP Wallonie |
| Albreht | Tit | National Institute of Public Health of Slovenia |
| ALLAOUI | EL MAATI | L’Agence InterMutualiste |
| Antoine-Poirel | Hélène | Sciensano |
| Apostolidis | Kathi | ECPC -European Cancer Patient Coalition |
| Arbyn | Marc | Sciensano |
| Balducci | Elisa | SPF Santé Publique |
| Bartoszek | Paulina | Cliniques Universitaires Saint-Luc, Institut IRAII |
| Bjerke | Astrid | Norwegian Cancer Society |
| Blondeel | Jean Pierre | Hodgkin en non-Hodgkin vzw |
| Boonen | Brigitta | Euroskin |
| Brems | Hilde | KU Leuven |
| Briers | Erik | Wij Ook Belgium # Europa Uomo - European Prostate Cancer Coalition |
| Brochez | Lieve | UGent |
| Broeckx | Tom | KULeuven |
| Burrion | Jean-benoit | BRUPREV - HUB/Institut Jules Bordet |
| Caillier | Marie | Centre de Psycho-Oncologie asbl |
| Candeur | Michel | CCR (Centre Communautaire de Référence pour le dépistage des cancers - Wallonie) |
| Ceratto | Nadia | MIUR |
| Chavez | Marcela | CHU de Liège |
| Coche | Emmanuel | Cliniques Universitaires St-Luc |
| Cokelaere | Kristof | jan yperman ziekenhuis |
| Colliez | Florence | Sanofi |
| Coosemans | An | KU Leuven |
| Daxhelet | Jean-Yves | MSD |
| De Cuyper | Astrid | UCL - Cliniques Universitaires St Luc |
| De Gauquier | Kristel | pharma.be |
| De Munter | Johan | UZ Gent |
| De Ridder | Karin | Sciensano |
| De Witte | Chloë | UZ Gent |
| Demoury | Claire | Sciensano |
| D'Hondt | Kathleen | VO- Dep EWI |
| Duyck | Valérie-Anne | O'YES ASBL |
| ERTAYLAN | GOKHAN | VITO |
| Fabri | Valérie | Union Nationale des Mutualités Socialistes - Solidaris |
| Fontaine | Christel | UZ Brussel |
| Francart | Julie | Belgian Cancer Registry |
| Francis | Laurent | UCLouvain |
| Froyen | Guy | Jessa ziekenhuis |
| Geboes | Karen | UZ Gent |
| Gijssels | Stefan | Patient Expert Center |
| Gilissen | Liesbeth | Centre of Environment and Health KU Leuven / IDEWE |
| Gils | Ann | Kom op tegen Kanker |
| HAGIEFSTRATIOU | Martini | Union Nationale des Mutualités Socialistes - Solidaris |
| Hernandez | Hegel | KU Leuven/Illumina |
| Janssens | Jaak | ECP |
| Jelenc | Marjetka | National Institute of Public Health |
| Kas | Koen | UGent |
| Kellen | Eliane | CvKO |
| Kridelka | Frédéric | CHU Liège |
| Kutlu | Funda | Cliniques universitaires Saint-Luc |
| Lahousse | Lies | UGent |
| Le Ray | Veronique | Foundation Against Cancer Belgium |
| Lefevre | Alexander | Roche |
| Lescart | Alexia | Commission communautaire commune |
| Lippens | Evi | UGent |
| Macq | Benoit | UCLouvain |
| Maes | Brigitte | Jessa Hospital |
| Martens | Patrick | CvKO |
| Martinive | Philippe | institut jules bordet |
| Merckx | Barbara | MSD |
| Montag | Ilke | Soc. Mut. |
| Nackaerts | Kristiaan | UZ Leuven /KU Leuven |
| Nogales | Luciana | ICON – Barcelona |
| Norga | Anita | Roche Diagnostics |
| Oculi | Celia | Roche |
| Olsen | Catharina | UZ Brussel |
| Padalko | Elizaveta | UZ Gent/Ugent |
| Paridaens | Henry | (CHR) Centre Regional de la Citadelle Liege |
| Parmentier | Marieke | Roche |
| Paye | Alexandra | CHU de Liège |
| Piel | Géraldine | Uliège |
| Pil | Lore | Kom op tegen Kanker |
| Poncin | William | Cliniques universitaires Saint-Luc |
| Rigolle | Cathy | Kom op tegen kanker |
| Schelfhout | Bart | Sanofi |
| Schreurs | Wim | Philips |
| Seriese | Iris | RIVM - National Institute for Public Health and the Environment |
| SIBILLE | Catherine | CHR Citadelle |
| Simoens | Cindy | Sciensano |
| Thibo | Johan |  |
| TOMBAL | Bertrand | Cliniques universitaires Saint Luc |
| Tuyaerts | Sandra | UZ Brussel |
| Urbina | Montse | FAPA |
| Van Damme | An | Cliniques Universitaires St Luc + BSPHO |
| Van de Walle | Liselot | Agentschap Zorg en Gezondheid |
| Van Den Bulcke | Marc | Sciensano |
| Van den Eynde | Sandra | Sensoa |
| Van den Eynde | Marc | Cliniques universitaires Saint Luc |
| Van der Heyden | Johan | Sciensano |
| Van Herck | Koen | Belgian Cancer Registry (BCR) |
| Van Meerbeeck | Jan | UZA |
| van Well | Maureen | pharma.be |
| Vandekeere | Saar | MSD Belgium |
| Vandevijvere | Stefanie | Sciensano |
| Volders | Pieter-Jan | Jessa Ziekenhuis |
| Westerlinck | Philippe | Private |
